# On the Psychology of Argument: A Structural Analysis of Former Muslims' Postings Within Malaysian Social Media

**DOI:** 10.3389/fpsyg.2021.740558

**Published:** 2021-10-22

**Authors:** Umair Munir Hashmi, Radzuwan Ab Rashid, Rabia Munir

**Affiliations:** ^1^Faculty of Languages and Communication, Sultan Zainal Abidin University, Kuala Terengganu, Malaysia; ^2^Faculty of Social Sciences, Sultan Zainal Abidin University, Kuala Terengganu, Malaysia

**Keywords:** Toulmin model of argument, Islamic teachings and practices, former Muslims' postings, social media, argument levels scheme, Malaysia

## Abstract

This research attempts to provide insights into the argumentation structures in the discussion of Islam on social media involving 14 Malaysian former Muslims. The social media accounts of the participants were observed for 12 months, from January to December 2019. A total of 368 postings put forth arguments related to Islamic authoritative discourse, the Quran and “Sunnah” of the Prophet Muhammad, to justify their renunciation of the Muslim religion. The analysis revealed that the Level 2 argument, which includes the claim, data to support the claim, and the warrant, was identified as the most common argument structure. Level 5, which has more than one rebuttal, was the least common argument structure. The analysis shows that most argument structures were at the lower levels (1–3) in that they offered no strong, clearly identifiable rebuttals. This study concludes that the arguments put forth by former Muslims, in the main, are loosely constructed rather than attempts to build a strong cumulative argumentation to support their reasons for abandoning the Muslim faith.

## Introduction

Social media has provided safe spaces offering freedom of expression to people who feel marginalized socially, politically, or religiously (Altoaimy, 2018). In addition, they have the opportunity to communicate and interact with friends and peers freely, due to the user's privilege to presume (produce and consume at the same time) opinions on social media. The most common social media such as Facebook, Twitter, and Instagram have shaped the narratives and practices of religious authority, religious identity, and religious community (Kgatle, 2018) where traditional offline religiosity is transformed through more subjective religious views and experiences (Campbell, 2012; Scardigno and Giuseppe, 2020).

The reflections from several recent studies have shown the use of social media as a multifunctional tool beyond the prevailing notion that social media is engaged merely for entertainment and information sharing (e.g., Kgatle, 2018; Tan Meng, 2019; Thomas et al., 2019; Hashmi et al., 2020; Scardigno and Giuseppe, 2020). Posting, sharing, liking, and commenting on religiosity have added to the functionality of social media such as creating online religious communities (Hashmi et al., 2020). Scardigno and Giuseppe (2020) pointed out that social media offered new spaces of confidence to the believers, which has attracted more subjective religious practices and the overt expression of personalized religious views. Performing religiosity within social media has become a dominant way of seeking satisfaction by the believers in Muslim majority countries (Thomas et al., 2019). Some believers anchor the religiosity within selected religious texts by invoking sacred text into their postings; some others invoke religious sermons by the preachers (Hashmi et al., 2020), whereas some cyber-believers are more flexible with the religious views (Campbell, 2012); this third category of the believers demonstrates mostly the personalized religiosity with decentralized interpretations and meanings of the religious teachings as compared to the set offline patterns of religiosity (Hashmi et al., 2020). Therefore, social media offers the users with online safe spaces that enable the users to express personalized views and interpretation of the religious teachings and subjective religious experiences, which is often a sensitive matter in the offline spaces (Tan Meng, 2019; Scardigno and Giuseppe, 2020; Hashmi, 2021).

On the other hand, believers' performance of traditional and culturally embedded religiosity is often contested by some social media users' flexible religion-related engagement and religion-related subjective opinions. According to Crawford (2002), people in different cultures have different practices and habits deeply embedded in their normative beliefs. There are also people from the same background who oppose, criticize, and challenge such normative beliefs and the practices associated with them in a particular culture. They may also engage in argumentation to disrupt or challenge these normative beliefs but the force of their argumentation and its persuasiveness depends upon the discursive space in which they construct their arguments. Taking on board Crawford's claim, we perceived the potential of finding argumentation examples in social media postings that criticize and challenge certain normative cultural beliefs and the religious practices associated with them in particular societies. This study aims at identifying and analyzing the argument structures in Malaysian former Muslims' postings on social media wherein they challenge Islamic authoritative discourse. Former Muslims in this study refers to individuals who were Muslims once, however, they lost faith in Islam and becoming an atheist (self-proclaimed) they reject all the religions that are claimed divine religions.

Malaysia is an Islamic country where several legal provisions restrict or otherwise criminalize alleged blasphemy to religion or religious figures, beliefs, or principles such as Section 298 and 298A (1) of the penal code, Section 3(1) and 4(1) of the Sedition Act 1948, Section 233 of the Communication and Multimedia Act (CMA), and Section 7(1) of the Printing Presses and Publications Act 1984. Section 233(1) (a) of the Communication and Multimedia Act is mostly deployed to criminalize alleged offenses on social media. It deals with inappropriate or offensive use of social media against those who allegedly insults Islam or Prophet Muhammad or any other religion as it proscribes “any comment, request, suggestions, or other communication which is obscene, indecent, false, menacing or offensive in character with intent to annoy, abuse, threaten, or harass another person”; and the punishment for this offense is up to one year's imprisonment, a RM 50,000 fine, or both (CMA, 1998, p. 78). For example, Malaysian authorities arrested Alister Cogia and convicted him under Section 298A of the Penal Code and Section 233(1) (a) of the Communications and Multimedia Act 1998 for posting offensive content on social media that was allegedly insulting to Islam and the Prophet Muhammad. He was sentenced to 10-year imprisonment and a RM 50,000 fine. His imprisonment was later on reduced to 6 years (Tawie, 2019). This shows how all Malaysians, including former Muslims or non-believers, have no offline safe space to express themselves and opine overtly in Malaysia due to the Malaysian Civil Law (Thaib, 2013; Hamid, 2017). Hence, in the past, before the advent of social media, dissenting Malaysians would have concealed any opinions and beliefs that were anti-Islamic (Mohamad et al., 2018). However, social media provides an opportunity for such closeted Malaysians to freely communicate, interact, and express their world views while masking their real identities. They post and comment on others' postings, not only to put forth their own views and beliefs of religion but to construct a discourse to justify and support their standpoint. Their postings are not simply a sharing of views; they try to use language strategically to make their postings logical. Researchers such as Mohamad et al. (2018) and Rashid et al. (2018) have pointed out that religiously marginalized people (e.g., former Muslims and non-believers) in Malaysia who appear on Facebook and Twitter use social media for several reasons such as entertainment, information dissemination, information seeking, seeking and providing social support, academic purposes, expressing their beliefs and standpoint concerning their renunciation of Islam, and criticizing Islamic teachings and Muslim beliefs. In order to justify their renunciation of the Muslim faith and to express their viewpoint, they construct arguments through their posts on social media. This study strives to examine the strategic construction of posts by former Malaysian Muslims on social media through the framework of the Toulmin Argument Pattern (TAP) and Erduran, Simon and Osborne (2004) argument-level scheme. TAP provides guidelines to help trace different argument structures, while the argument-level scheme focuses on the strength of the components of argument, whereby clearly identifiable rebuttal is considered the strongest form, and an argument with more than one such rebuttal is considered the most forceful argument. Toulmin model of argument structure is an old-fashioned model but it has been enjoying a consistent revival over time because of its utility in the research studies on argumentation. Several researchers such as Metaxas et al. (2016), Kathpalia and and See (2016), Pedemonte and Balacheff (2016), Moon et al. (2017), and Drury et al. (2019) used the Toulmin model to identify weaknesses and strengths of argument structures in the context of students' classroom discussions.

## Theoretical Framework

### Toulmin's Model of Argumentation

**Figure 1:**
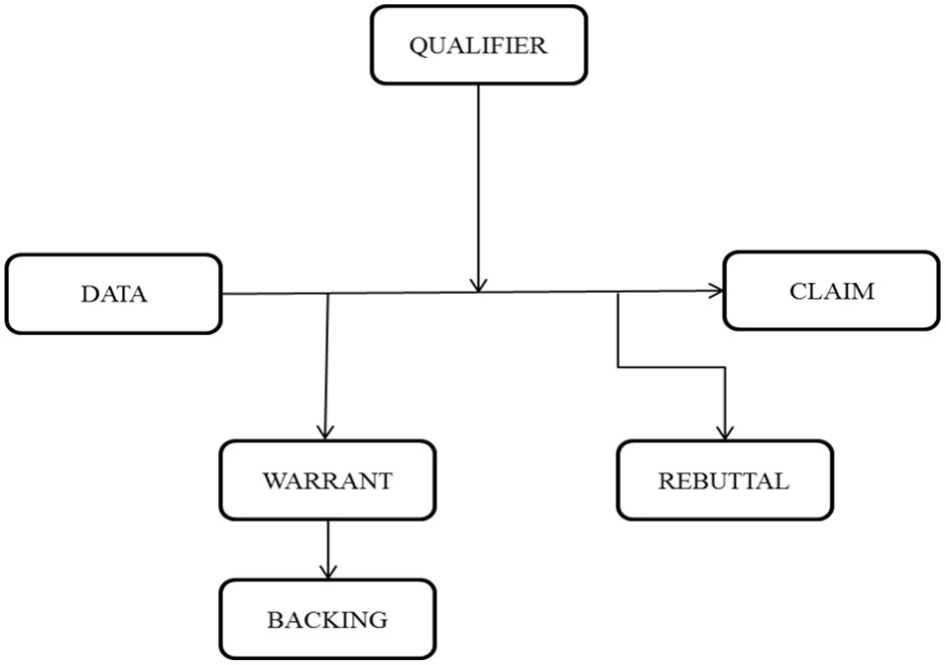
Toulmin's model of argument (Toulmin, 1958, p. 97). This figure summarizes the theoretical perspective employed in analyzing the data.

Toulmin (1958) proposed six components of argumentation; the first triad (Claim, Data, and Warrant) is crucial for the basic formation of an argument, while the second triad (Backing, Rebuttal, and Qualifier) is sometimes explicit, but mostly implicit in the argument. In either case, it strengthens the argument and tends to extend it (Toulmin, 1958). Toulmin (1958) illustrates the functioning of each component in this model as shown in Figure 1.

#### Claim

A claim is an assertive statement that embodies the main purpose of the argument (Toulmin, 1958).

#### Data

The information offered as the basis for the argument is called data (Toulmin, 1958). In some scenarios, additional claims are made to support the central claim of the argument; such claims function as data for the main claim (Hoeken et al., 2012).

#### Warrant

The most important element of the Toulmin model is the warrant; the statement that is used as a standard, concept, principle, or license for a conclusion; it functions as a link between the data and the claim. The warrant is an implicit or explicit rule or grounds that demonstrate the validity of the data to the claim (Toulmin, 1958). Sometimes, there is no need for additional criteria in arguments to distinguish between what counts as data and warrants. In this situation, implicit details can be used to distinguish between data and warrants in a particular argument (Toulmin, 1958; Simosi, 2003). Each assertion is considered part of the evidence for the argument in question when a statement is used to interpret the condition/situation concerned. To show the importance of certain information to the argument when different arguers use the (same or similar) statements, these statements take on the role of warrant in that specific case.

#### Backing

According to Toulmin (1958), an inference that supports the warrant is called *backing*. The backing can be a piece of factual information (e.g., previous observations) or a concept, value, or opinion, which is derived from past experiences or perceptions in the context of the arguer's personal or institutional background.

#### Rebuttal

Toulmin (1958) describes rebuttal as a statement addressing any limitations to the claim, which may genuinely apply, e.g., exceptions. Finocchiaro (2012) further explains that rebuttals can have several functions in the Toulmin model. For example, rebuttals may identify circumstances in which the authority of a particular warrant should be put aside. Hence, a rebuttal provides exceptions to the applicability of the warrant or limits the generalizability of the warrant and claim.

#### Qualifier

The qualifier shows how good the data bound into the warrant is, and to what extent it can restrict the universal application of the specific claim (Toulmin, 1958). Phrases or words conveying the author/speaker's level of certainty regarding their claim are identified as qualifiers in the Toulmin model of argument analysis (i.e., hedges such as some, most, maybe, almost, probably, likely, certainly, and apparently).

## Recent Studies on Argument Analysis Using Toulmin's Argument Pattern

Toulmin's (2003) theory of argument patterns provides a clear process for the evaluation of arguments (Metaxas et al., 2016). The integration of Toulmin's model with other analytical approaches such as the argument-level scheme has the potential to lead to the ability to define many facets of argument in different fields (Simon, 2008). Several researchers have utilized Toulmin's model of argumentation in their studies to analyze and illustrate different aspects of argument (e.g., Simosi, 2003; Simon, 2008; Hoeken et al., 2012; Kathpalia and and See, 2016; Metaxas et al., 2016; Pedemonte and Balacheff, 2016; Moon et al., 2017; Drury et al., 2019).

Kathpalia and and See (2016) focused on 100 university student blogs written before instruction concerning argumentation and after instruction on argumentation during a classroom writing course. The results showed that more than half of the students improved their argument structures and the quality of their argumentation in the blogs written after receiving instruction about using the Toulmin model. Pedemonte and Balacheff (2016) evaluated the role of individual conceptions for evidence-building in Mathematics education by designing an enriched model based on the Toulmin scheme of integration to analyze the relationship between the warrant and backing of the claim in mathematical problem-solving. Moon et al. (2017) analyzed students' argumentation in a classroom setting through the Process-Oriented Guided Inquiry Learning (POGIL) approach using the Toulmin model of argumentation. The researchers identified two argumentation objectives: persuasion and consensus, in the argumentation of students. The findings showed that most students were able to construct persuasion arguments with two-component structures, whereas consensus two-sided argument structures lacked additional rebuttals (Moon et al., 2017).

Erduran, Simon and Osborne (2004) research report illustrates the analytical affordance of Toulmin (1958) argument pattern (TAP) in the analysis of argumentative discourse in science classrooms. This research report is based on a longitudinal research project “Enhancing the Quality of Argument in School Science” carried out from 1999 to 2002, so focused on classroom interactional discourse and the dynamics for initiating and sustaining argumentation. Erduran et al. (2004) proposed two methodological approaches for the analysis of interactional argumentative discourse. The first deals with TAP's usefulness for quantifying arguments generated in the context of teacher-students' whole-class discussions. It also provides some qualitative insights into argumentation by comparing the dynamics of the arguments generated in different lessons over time. The second approach deals with TAP as a measure of students' use of rebuttals in small group discussions. Rather than using TAP as a statistical tool, the researchers adapted the technique and applied TAP to the same individual lesson taught by the same teacher in two successive years. The researchers drew TAP profiles for each teacher's discourse practices while conducting the same lessons in year one and in the following year, which indicated whether the teachers had improved the quality of their argumentation during the same lesson in the following year. The comparisons of the teachers' TAP profiles showed that they were similar for the same lesson—the first taught in the first research year and the other in the second research year. But the TAP profiles of the individual teachers were different. Such qualitative comparisons showed how the teachers' argumentation in classroom discourse differed and which aspects of the discourse needed corrections to improve the quality of the argumentation.

Erduran, Simon and Osborne (2004) argument-level scheme mainly focused on the rebuttals and the strength with which the students countered each other's arguments. According to this system, the strength of the argument depends on the presence or absence of rebuttals as well as the nature of the rebuttals, whether weak rebuttal or clearly identifiable strong rebuttals. Erduran, Simon and Osborne (2004) study showed the methodological potential of TAP as an indicator of the quality and quantity of the arguments in classroom discourse.

## Recent Studies on Leaving Religion With a Special Reference to Islam

Enstedt et al. (2019) argue that the question of what “leaving religion” entails is difficult to answer precisely in isolation because the process of leaving religion involves different social, political, cultural, and religious factors. Though, former Muslims may offer and express blatant critique due to their own lived pathetic experiences yet some social norms and practices that are deeply influenced by Islam continue to shape their life patterns (Larsson, 2018; van Nieuwkerk, 2018). In an academic study of religion, “apostasy” is considered an appropriate equivalent of “leaving religion.” Lewis (1993), a renowned scholar of Islamic studies, argues that “apostasy” entails “leaving Islam” by overtly declaring it or it is conferred by the Muslim community and religious authorities upon a person who denies any of the fundamental beliefs in Islam such as Quran and Sunnah of Prophet Muhammad. Islamic exegetes Imam Ahmad Ibn Hanbal, Imam Abu Hanifah, Imam Malik, and Imam Shafie agreed upon the capital punishment for apostasy (Ismail and Awang Mat, 2016), whereas in view of several classical Islamic jurists such as Ibrahim Al-Nakhaie (d. 95 A.H), Sufyan Al-Thauri (d. 162 A.H), Al-Tabari (d. 923), Ibn Taymiyyah (d. 1328), and Al-Shawkani (d. 1834), apostasy in Islamic traditions is socially a crime only if it is accompanied by the treason against the community, leadership or the state; otherwise, it is religiously an obnoxious sin for which there is no temporal capital punishment (see El-Awa, 1993; Akhtar S., 2011; Ismail and Awang Mat, 2016). “Sin,” in this context, entails that the apostate is dammed and doomed in this world and the world hereafter (Hamid, 2017). In the contemporary Muslim majority countries, an apostate is considered a treacherous and traitor of the community in which he/she enjoyed loyal relations, participation, and acceptance as a member, for which he/she becomes “a dead limb to be excised” (Lewis, 1995, p. 229). The above discussion shows that leaving Islam in the individual capacity is not a temporal crime for which state authorities can intervene unless the apostate tries to commit or ignite treason.

The term “former Muslim” entails that a person who once believed in the Muslim faith, Islam, and then renounced his/her faith in Islam is considered an apostate who is liable to face conviction and legal consequences in the Muslim majority countries (Lewis, 1993; Warak, 2003). To understand the Malaysian former Muslims' criticism of the religion, it is necessary to briefly contextualize the laws of apostasy in Malaysia. Malaysia is an Islamic country with a federal constitutional monarchy. Nine states have their respective hereditary rulers where the respective rulers of Selangor, Terengganu, Kelantan, Pahang, Johor, Kedah, and Perak are constitutionally recognized as sultans; the rulers of Perlis and Negeri Sembilan are recognized as Raja and Yang Di Pertuan Besar, respectively, whereas, the rulers of the states of Penang, Malacca, Sarawak, and Sabah are appointed by the Yang di-Pertuan Agong (Head of the State) for a period of 4 years and are constitutionally recognized as Yang di-Pertua Negeri. These 13 rulers frame the constitutional monarchy in Malaysia (Article 38(1) Constitution 2003). The nine hereditary rulers have the constitutional provisions of electing among themselves, a Yang di-Pertuan Agong (Head of the State) for the reign of 5 years on rotation based system. The sultans are the head of Islam in their respective states that adds the characteristics of Islamic monarchy to the constitutional monarchy in Malaysia (Hamid, 2017). For the status of “the head of Islam,” they are highly revered in their states. Malaysian Constitution ensures the special status of the monarchy. Loyalty to the King and the rulers of the states becomes a tradition and custom to the Malay community. The constitution bestows special privileges to the monarchy in Malaysia such as appointing judges, civil servants, 40 members of parliament, etc. (Federal Constitution 1957., 2010). Being head of Islam, the rulers of nine states have immunity from any type of criticism. They have a powerful role in establishing, preserving, and ensuring the implementation of sharia laws in the country (Fernando, 2006; Abdullah, 2009).

There are two types of former Muslims in Malaysia: the apostates who were Malay born and bred and the apostate who were converted Muslims. The Malays enjoy a special and privileged status in Malaysia, whereby Article 160 (2) of the Federal Constitution of Malaysia (1957) states that “Malay means a person who professes the religion of Islam, habitually speaks the Malay language, confirms to Malay custom and, *(a)* was before Merdeka Day born in the Federation or in Singapore or born of parents one of whom was born was born in the Federation or in Singapore, or is on that day domiciled in the Federation or in Singapore; or *(b)* is the issue of such a person” (p. 142). Being constitutionally a Muslim, rejection of the faith by a Malay person is considered the most heinous crime legally and socially compared to the former Muslims who were new converts formerly (Mohamad et al., 2017; Hamid, 2018). To avoid conviction under Islamic laws in Malaysia, Malaysian citizens who want to leave Islam to need to get confirmation from the “Shariah court,” which has jurisdiction of Islamic laws (Hamid, 2017, 2018; Salleh et al., 2017; Hashmi et al., 2020). The consequences of leaving Islam in Malaysia include dissolution of marriage, distribution of the jointly-acquired property during the marriage, cancelation of the Malay title of the land, and the revocation of children's guardianship and custody (Dahlan and Faudzi, 2016; Ismail and Awang Mat, 2016; Ismail and Al-Subaihi, 2020). Several studies (e.g., Fernando, 2006; Abdullah, 2009; Shuaib, 2012; Daniels, 2017) pointed out that Shariah laws and the jurisdiction of Shariah court are demarcated to Muslims only, but Shariah court more or less has been under the influence of the agenda of political Islam by the main political parties. Malaysian Islamic Development Department [JAKIM] has been a subject of severe criticism by the human rights activists in Malaysia, mainly due to its allegedly ultra-Islamic stance on Lesbian, Gay, Bi-sexual, and Transsexuals (LGBTs), apostates, and the critics of political Islam in Malaysia (Zulkffli and Ab Rashid, 2016, 2019). LGBT hold the institutionalized discrimination and misconduct toward them, responsible for their severe critique of the religion in Malaysia mainly due to the JAKIM's criminalization of non-normative gender expression (Tamilchelvan and Rashid, 2017; Ghoshal, 2021). Another vital factor that invited severe criticism of the religion is the confusion of the jurisdiction of Federal court and that of Shariah court, which has made the procedure for application to leave Islam impractical in spite of the fact that the Federal Constitution 1957 (2003) ensures that civil law is superior to the sharia law (Abdullah, 2009; Human Rights Watch., 2014; Ismail and Awang Mat, 2016). In light of the above discussion, this study argues that the former Muslims cannot overtly express themselves and are criminalized in Muslim majority countries. Human Rights Watch. (2014) has also reported that LGBTs have long been denied their right to free choice and freedom of speech due to the supremacy of political Islam in Malaysia. That is why, in the online safe spaces of social media, they structure arguments to express their viewpoint attempting to justify their criticism of the religion and Shariah laws. This study adopts a purely linguistic perspective of the argumentation to analyze former Muslims' argument structures within their social media postings. The objective of this study is neither to contest former Muslims' anti-religion discourse and nor to reject the justifications of renouncing Islam; rather, it attempts to provide insights into how the necessary linguistic units of one's stance can be identified and sequenced to construct a high level of argument structure to achieve linguistically attractive and complete micro-argumentation in the limited space of micro-blogging within social media.

## Methodology

This study took a qualitative approach to identify and analyze the argument structures in the social media postings of former Muslims. Facebook with 91.7% and Twitter with 37.1% of Malaysian users are two famous social media platforms for content sharing in Malaysia (Malaysian Multimedia and Communication Commission [MCMC], 2020). Fourteen social media users who self-identified as former Muslims born and brought up in Malaysia as Muslims, and later on renounced Islam and declared that they were atheists on social media, were recruited to participate in this study. For this purpose, Malaysian social media users who had managed to seek asylum abroad after facing many death threats in Malaysia due to their rejection of Islam, and use their real identity on the site, were identified. Using the snowball technique, the potential participants were identified. All of them were contacted to seek their informed consent.

To ensure and assure all the participants of their protection, the researchers took necessary measures such as using pseudonyms for all 14 participants and removing all personal data from the excerpts presented in this article. This study argues that the former Muslims are not hiding within social media, and they have been postings for years. The construction of their postings is strategic enough to avoid being considered criminal; that is why the authorities have not shut down their social media accounts. With the consent of the participants, their social media postings in the back-dates from January to December 2019 were observed to avoid the potential desirability of being a part of this study and to ensure the generation of data from naturally occurred postings by the participants.

The participants put more than 2,200 posts on Facebook pages and Twitter groups. These posts consisted of a variety of topics ranging from social, cultural, political to personal opinions. Most of the time, posts were retweeted and shared by other members of the community. However, 368 posts discussed the Islamic authoritative discourse (Quran and Sunnah of the prophet Muhammad) that serves as the data for this research article.

## Selection and Analytical Process

In the first phase, the thematic scrutiny of the data was undergone to distribute it under different topical categories such as challenging worship practices, challenging authenticity of Islamic authoritative discourse, criticizing traditional Muslim women, endorsing Theory of Evolution, challenging the criminalization of LGBTs, expressing Atheism, proving Islam as a religion of extremism, and demanding freedom of speech. We selected the postings for presentation based on degradations and contrasts among all the postings. The postings made by Malaysian former Muslims, and frequently shared and re-tweeted by the former Muslims from other nationalities; comparatively more insightful postings; and in contrast to the implicit expressions, the postings explicitly challenge the religion was chosen for the presentation. As an example, we explain the selection process of Adams' Tweet 11 that explicitly rejects Islamic authoritative discourse on LGBTs, criticizes Malay laws of criminalizing LGBTs, and urges legal reforms. Out of the 54 postings touching on the topic of LGBTs, Adams post was identified as unique due to its meticulous content and potential of the serious challenge as Adams informs that he works in the global context of criminalizing LGBTs, implying that the scope of his challenge is not limited to Malaysia only and that makes this posting unique among the other postings expressing personal experiences and with limited scope. In the fashion of discourse papers, to avoid length constraints, it is a common practice to present selected extracts from a large data because the presentation of the whole analyzed data takes up lots of space. To ensure the validity of the findings, the topical categorization was reconfirmed by the two co-authors, whereas the findings were discussed with an inter-coder, a senior researcher in the field of argumentation; he agreed that there were overlaps of argument structure in the identified postings, which helped us to identify the major structure and level of argument in each of the postings and then add to the findings.

The data analysis involved the Toulmin model of argumentation to identify the argument structures in the postings and simultaneously Erduran, Simon and Osborne (2004) analytical lens were used to analyze the level of the argumentation. Erduran, Simon and Osborne (2004) framework is based on the Toulmin model, which categorizes argument structures into five levels.

1. ***Level 1*** argumentation has a simple claim against a counterclaim, a claim against a claim.
2. ***Level 2*** argumentation consists of a claim with data, warrants, and backing but lacks any rebuttal or refutation of a claim.
3. ***Level 3*** arguments have a series of claims supported by data, warrants, or backing commonly or separately, including the occasional weak rebuttal.
4. ***Level 4*** arguments have a claim with a clearly identifiable rebuttal and are considered strong arguments. Level four arguments may contain within them a series of claims and counterclaims.
5. ***Level 5*** argumentation is the strongest type, displaying an extended argument with two or more rebuttals.

(Erduran et al., 2004, p. 928)

Codes were assigned to the participants' postings on the respective social media platforms, Twitter and Facebook. Examples of the codes are as follows:

1. [Zik, FB. 7] refers to the seventh Facebook posting by Zik in the data set.
2. [Aris, T. 153] stands for Tweet 153 by Aris in the data set.

## Analysis and Discussion

**Figure 2:**
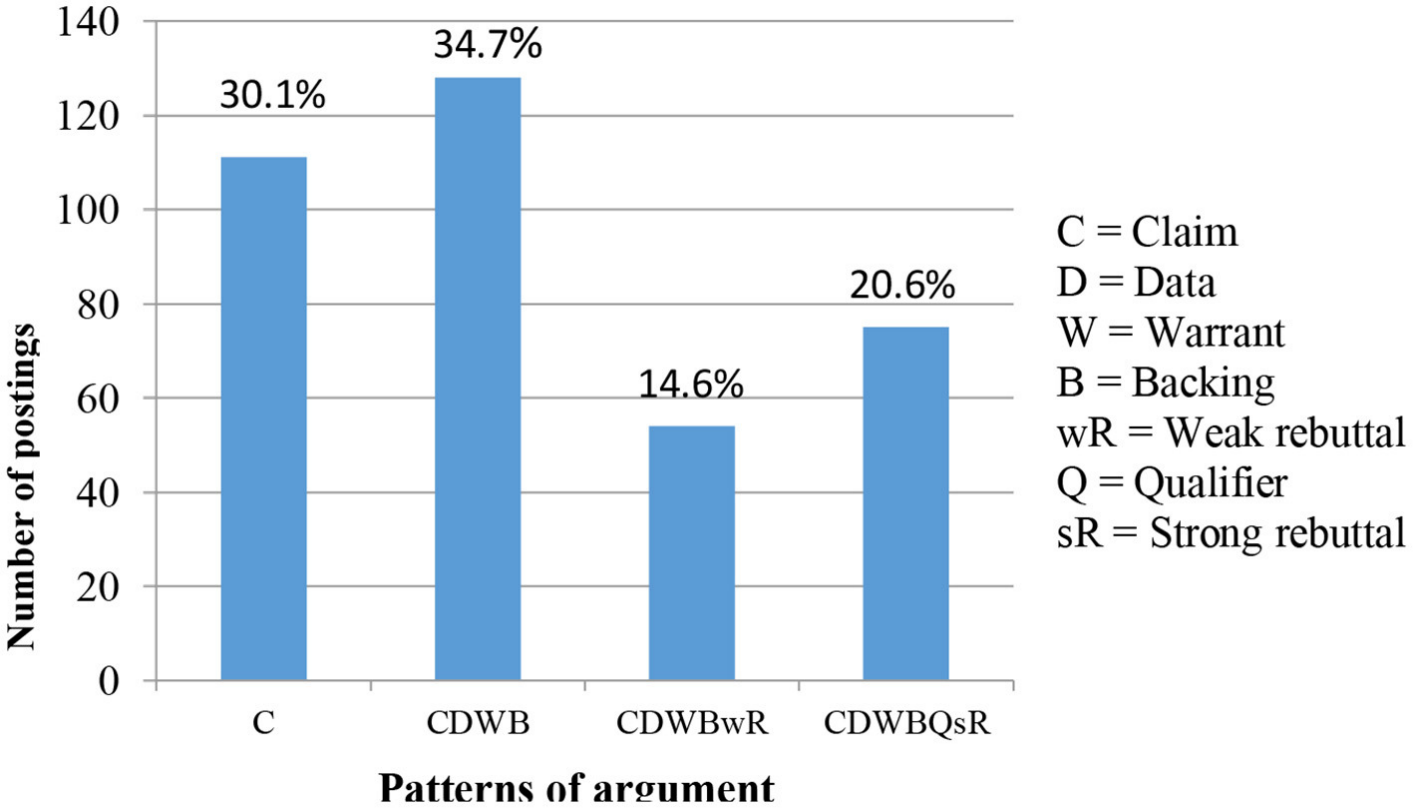
The percentage of the argument patterns. This figure shows the percentage of the argument patterns identified in this study.

The researchers applied the Toulmin model of argumentation deductively to the social media posts in which the participants challenged Islamic authoritative discourse that is Quran (Holy book of the Muslims) and Sunnah (traditions set by Prophet Muhammad). Figure 2 presents an overview of the identified argument structures in the participants' postings. In this graphical illustration, the percentages indicate the overall occurrence of each argument pattern identified in the data.

The percentage of strong arguments (i.e., Levels 4 and 5) is combined in the fourth bar in the above diagram (20.6%). In contrast, the first three bars show the different structures of the weaker argumentation. The following are examples of different argument structures identified in the participants' social media postings, and their respective argument levels, according to Erduran, Simon and Osborne (2004) argument-level scheme.

### Extract 1

1. Having lived as a Muslim I have known it as a strategy of Muslims
2. that good Hadith proves the greatness of Muhammad and
3. bad Hadith is always declared to be weak and nonfactual.
4. Similarly, good parts of the Quran are considered proof of the greatness of Islam
5. whereas, bad parts are always justified by saying that they have been taken out of context and
6. as a result, you are declared an islamophobe.

[Zik, FP. 6]

The above extract is part of an argument challenging Islamic authoritative discourse, which claims that the Quran and the Hadith of the Prophet Muhammad are two flawless sources containing a complete code of behavior for humanity. In Islamic terminology, Hadith is a “report of the words and deeds of Muhammad and other early Muslims; it is considered an authoritative source of revelation, and it stands second only to the Quran” (Oxford Islamic Studies Online, 2021). The argumentation in this extract consists of assertive statements which Toulmin (1958) uses to refer to claims as components of argumentation. Hence, the argumentation traced in this extract is a series of claims without any supporting data or warrant. The first claim (line 2) and the second claim (line 3) together challenge the flawlessness of Muhammad's Hadiths. The flawlessness of the Quran is challenged in the third and fourth claims (lines 4 and 5). Past experiences and observations to support the main claim or the warrant are considered as “backing” from the perspective of Toulmin's argument pattern (Simosi, 2003; Simon, 2008). In line with this proposition, the opening part of the argument (line 1) is considered as weak backing for these claims. The backing is considered weak because it is not a shared experience and cannot be generalized. Line 6 presents a fifth claim, which concludes the argument but is different from the four other claims as it refers to the Muslim claim of Islamophobia.

In order to see the qualitative strength of such argumentation, we applied the argument-level scheme proposed by Erduran et al. (2004). The argument based on a simple claim or series of claims arguing against another claim, counterclaim, or series of claims, is considered to be a Level 1 argument (Erduran et al., 2004). Thus in light of the argument-level scheme, Extract 1 provides a Level 1 argument, which, according to Erduran et al. (2004), demonstrates the weakest frame of argumentation.

The following extract is a Tweet by Lily (pseudonym) in which she argues about women's oppression. The nature of its structure is similar to the argumentation presented in Extract 1.

### Extract 2

1. It's always women of privilege who talk the loudest about how Islam never oppresses women.

[Lily, T. 75]

Extract 2 presents an assertive statement, which according to Toulmin (1958) is a claim in argument terms. The claim in this argument serves as a counterclaim because it challenges Muslim claims that Islam protects women's rights. In this way, the argument in this extract put forwards a counterclaim challenging an earlier claim (Islam protects women's rights), which according to Erduran et al. (2004), is a Level 1 argument (i.e., the weakest form of argument due to the absence of data, warrant and backing). The following extract presents a social media posting by Hubert in which he argues against the creation theory of Islam and defends Darwin's Theory of Evolution.

### Extract 3

1. If you were tasked with designing the human eye completely from scratch, would you have purposely incorporated a blind spot?
2. Octopus eyes evolved separately from human eyes, and because of this, octopuses don't really have a blind spot.
3. A truly great example of convergent evolution.

[Hubert, FP. 94]

The argumentation in Extract 3 consists of a claim, data, and warrant. Toulmin's model requires researchers to look deeply into argumentation to trace the implicit components of the argument (Simosi, 2003). In line with this, the data (line 2) and warrant (line 3) implicitly put forward a claim (line 1), which can be identified as the *blind spot (an imperfection in the human eye cannot have been created on purpose*. Islamic authoritative discourse claims that God is the creator of everything and all his creations are perfect. In the light of the creationist theory of Islam, the counterclaim from the above argumentation can be boiled down to *God is not the creator of everything today –creatures evolved in different ways over time*. The data (line 2) supports this claim well and the warrant (line 3) embarks on the strong relationship between data and claim by introducing the license of “convergent evolution” to explain differences. Based on Toulmin's (2003) model of argument, three components, claim, data, and a warrant can be identified in the structure of argumentation in Extract 3.

Level 2 argument consists of a claim supported by data and a warrant (Erduran et al., 2004). This level argument is considered a common argument as it embodies the first triad of components (Toulmin, 1958; Erduran et al., 2004). According to Erduran et al. (2004), the argument presented in Extract 3 measures up to the requirements of a Level 2 argument and is considered a common argument form, lacking strong rebuttal. The following extract demonstrates a short argument by Adams. He challenges Islamic teachings and Muslims' behavior toward LGBTs.

### Extract 4

1. Being LGBT cannot be a crime.
2. Humanity is diverse,
3. Tolerance of difference is needed
4. and respect for all.
5. We cooperate globally to reform the laws where being LGBT is still considered a crime.
6. We would like to engage with Malaysia toward achieving this goal.

[Adams, T. 11]

Extract 4 offers an argument consisting of a general, explicit counterclaim (line 1) that challenges the Islamic authoritative discourse on LGBT. According to Toulmin (1958), universal truth provides a license for a relevant persuasive statement and is considered as a warrant in his argumentation pattern. “Humanity is diverse” (line 2) is a universal truth and can be categorized as a warrant for the data (lines 3 and 4). The argument structure then provides a backing statement (line 5), which serves as factual information to support the warrant (Toulmin, 1958). Finally, the arguer presents an implicit claim (line 6) that is specific, unlike the general claim (line 1). The implicit specific claim in line 6 can be glossed as “Being LGBT is illegal, a crime in Malaysia so, Malay laws about LGBT need to be reformed.” In line with Toulmin's (2003) model of argumentation, the argument in Extract 4 has four components: general and specific claims, data, warrant, and backing.

According to Erduran et al. (2004), an argument containing claim(s) with supporting data, warrant, and backing can also be considered a Level 2 argument, a common argument frame, lacking the contextual qualitative strength infused by the rebuttal, such as the context in which the claim stands being strong and with no exceptions where the claim does not apply (Erduran et al., 2004). The following extract is a social media posting by Sonia. She defends the atheists' point of view and their rights in this argument.

### Extract 5

1. Atheists are wrongly considered arrogant for rejecting funny and absurd stories from Islam like the
2. the world was created in 7 days; the first woman was made from Adam's rib and ate magic fruit from Satan; a couple of each animal species in the world went to the Middle East to get on a boat made by an 800-year-old man etc.
3. It is absurd that if you believe in such stories it's ok, otherwise you are arrogant because you ignore the realities and prefer your own opinion.
4. But atheists are arrogant in some ways, I won't deny it.

[Sonia, FP. 139]

Islamic authoritative discourse asserts that all those who do not believe in God are arrogant due to their ignorance. Sonia put forwards a counterargument to challenge this specific authoritative discourse. The assertive statement (line 1) is an explicit counterclaim, which is followed by data (line 2) and a strong warrant (line 3). According to Toulmin (1958), a rebuttal is a word or statement that presents exceptions to the claim or expresses the limitations of the claim. The strength of the warrant and backing is also affected if limits are placed on the claim under certain circumstances (Simosi, 2003). In the above-mentioned argument, a weak rebuttal is presented (line 4). It is considered a weak rebuttal because it does not offer clear exceptions or limitations to the claim.

According to Erduran et al. (2004), argumentation with one or more claims supported with data, a warrant, and a weak rebuttal, demonstrates Level 3 argument. In light of this proposition, the argument structure identified in Extract 5 fulfills the requirements of a Level 3 argument. Erduran et al. (2004) say that a Level 3 argument has a stronger persuasive quality than a Level 2 argument. The following extract presents a posting from Kris. It is a long argument challenging Islamic authoritative discourse on the Muslims' call to prayer (Adhan) (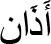) that is recited on loudspeakers five times a day to invite the Muslims to join prayer with other Muslims at the mosques.

### Extract 6

1. You know, just because most people don't complain about the call to prayer (Adzhan) and because of that you think “it doesn't bother people”, that doesn't necessarily mean that it's “really not” (a bother).
2. I know that there are people who are dissatisfied with how loud Adzhan is, especially in a Muslim majority country like Malaysia, but they're not brave enough (and it's understandable) to complain, because that would count as “blasphemy against Islam”.
3. A few rare cases have happened related to this, where a person complained that the Adzhan is too loud,
4. and it bothers people especially those who want to sleep and to rest, and what's really unfortunate is when their house is very near the mosque.
5. Sometimes the Adzhan that comes from the mosque can use maximum volume for literally no reason.
6. I too have experienced this in my younger days. But I didn't have the guts to say anything against Adzhan, although deep down, it annoys me, especially when I'm about to sleep and have a rest.
7. We live in an era where we can install apps that remind you of the call to prayer and the exact time for the five times a day prayers. Isn't that enough?
8. On the other hand, I have nothing against Adzhan when it's Friday prayers, but please don't use maximum volume for no reason.

[Kris, FP. 218]

This long argument consists of two claims (lines 1 and 5) supported by data (lines 2 and 4). The relationship between the data and claims is justified with a strong warrant (line 7), and backing (lines 3 and 6) has been provided to enhance the supportive potential of the warrant. The arguer also uses qualifiers *few, rare* (line 3) and *sometimes* (line 5), which brings clarity to the scope of the claims. The argument concludes with a weak rebuttal (line 8) in which the arguer offers the exception of his claim for the call to Friday prayer. The rebuttal in this argument is considered weak because the whole argument revolves around the disturbance created by the call to prayer but the arguer does not clarify why he excludes from rebuttal the remaining calls to prayer four times a day. This complicated argument does not measure up to Toulmin's (2003) pattern of argument. Although the arguer presents a claim (line 1) and provides data (line 2), the observation and experience-based statements (lines 3 and 6) are essentially the “backing,” which, according to Toulmin, should be provided after the warrant in order to support it. However, in this argument, the warrant (line 7) comes after the backing statements, which becomes a separate claim without a warrant and disrupts the whole argument.

At the same time, analyzing the argument using Erduran, Simon and Osborne (2004) lens of argument levels, it is a Level 3 argument, similar to the argument presented in Extract 5. In spite of the similarity of components, the argument in Extract 6 is different and weaker than the argument identified in Extract 5 in terms of its pattern.

Azurey is the arguer in the argument presented as Extract 7. She challenges the Muslims' claim that Islamic teachings are for promoting harmony and peace.

### Extract 7

1. You claim harmony and peace
2. but your Quran teaches not to accept others except those having the same belief as yours,
3. otherwise kill them.
4. You say that the Bible is a corrupted book
5. sometimes you say most of the Bible is corrupted
6. and still, you use its references to prove your beliefs.
7. how do we know which part of the bible is corrupted or not? >basically, if a particular chapter in the bible mentions the prophet Muhammad, that chapter is not corrupted.
8. Everything else is corrupted.
9. You check the Bible in light of the Quran but not vice versa.
10. You say some parts of the Bible are correct. hmmm what an accurate method of determining which part of the bible is corrupted. ^*^applause^*^.
11. And if one says most parts of the Quran are against human rights, and in order to prove it wrong, you are taught to kill the people.
12. It is ok some of the verses support women and human rights but most are dangerous to humanity.
13. Why don't you follow and preach what supports human rights for the sake of harmony and peace? Isn't it in the Quran?

[Azurey, FP. 255]

From Toulmin's (2003) perspective, the argumentation in Extract 7 contains all the key components of an argument. In the series of claims, the first counterclaim (line, 2) challenges the Quranic teachings and the Muslims' belief that the Quran teaches harmony and peace (line 1). The data (line 3) partially explains that Muslims are taught to kill non-believers. It implicitly refers to the early Islamic concept of Jizyah as the early history of Islam witnessed that non-believers were invited to embrace Islam or to pay a tax (Jizyah), where the purpose of jizyah was made clear in the light of the Quran, Surah Al-Taubah verse 29, stating that against the paid jizyah, the non-Muslims are given life protection in an Islamic state such as security of their lives and property, comfort and convenience in dealing with Muslims, and social welfare (Ghozali and Nugroho, 2021). Sometimes the warrant is hypothesized as generally known to others and is left implicit; such implicit reasoning must be taken into account in analyzing argument structure (Simosi, 2003). The researchers here consider this implied information as the warrant. On the one hand, Muslims' claims about the Bible (lines 4 and 5) provide backing for the counterclaim (line 2) that the Quran teaches not to accept others, rather than preaching harmony. On the other hand, there is the claim (lines 4 and 5) of contradiction in Muslims' beliefs. This contradiction is elaborated with data (line 6) that a corrupt book cannot be used to support beliefs. The warrant (line 9) supports the data by confirming that the parts of the Bible which support Muslim beliefs are correct. The backing (lines 7 and 8) present an example, which is the most quoted by Muslim scholars, that Muhammad was mentioned in the Bible (see Dawud, 1978; Badawi, 2005), so this part of the Bible is correct, but everything else in the Bible has been corrupted. The satirical statement (line 10) also extends the backing. In terms of the counterclaim (line 2) that the Quran does not teach harmony and peace, a strong warrant (line 11) points out that Muslims are free to declare the Bible a corrupted book, but someone points out the verses of the Quran that are against human rights, Muslims are taught to kill them to prove them wrong. In light of the above data, warrant and backing another claim is leveled in line 12, which says that most of the verses of the Quran are dangerous to humanity. A strong rebuttal (line 13) agrees that Quran has some parts that support human rights wherein the qualifier *some* limits the generalization of the rebuttal. In this way, the limitation of the claims and counterclaims is expressed through a rebuttal. At the same time, the rebuttal paves the way for suggesting that harmony and peace can be achieved by Muslims through preaching and focusing on the teachings of the Quran which believers and non-believers have in common. According to Stapleton and Wu (2015), the argument with strong rebuttal is perceived as highly persuasive and ranked as having a high argumentation profile. The argument in this post is a strong persuasive argument.

The researchers then applied Erduran, Simon and Osborne (2004) argument-level scheme, which claims that an argument consisting of several claims and counterclaims supported with data and warrant, and with a strong rebuttal is a Level 4 argument, a strong argument (Erduran et al., 2004), which can have extended argumentation if comparatively more robust rebuttals are provided (Simon, 2008). The following extract shows Cathy's post on Facebook in which she challenges the Islamic teachings about leaving Islam and argues strongly for equal human rights, especially in Malaysia; a country where Islamic laws are enforced.

### Extract 8

1. It's sad to see that there is no realistic individual freedom for Malays in Malaysia to
2. question their faith, to convert, or to leave religions altogether.
3. The fact that the majority of Malay Muslims are indeed fine (with the fact) that they are not granted
4. individual freedom and strictly prohibited from thinking freely for themselves
5. because they seek help from Islamic scholars on whatever issue there is, including
6. science and mental health
7. which is incredibly worrying and manipulative.
8. Years of brainwashing people are the cruelest thing you can do to an individual
9. who was born as a person who knew nothing at first (was innocent).
10. To see that other people enjoy freedom, while the Malays are prohibited from it
11. and the fact that the majority of them have no problem about being trapped and judged
12. under the name of the so-called characteristics of how to define a Malay
13. is the major key point that people need to start to realize how cancerous it is.
14. Once they are given freedom and the realization that there are many faiths and religions and still want to follow Islam, it is their right and we, the free thinkers, will respect their choice
15. if they don't threaten others' freedom.

[Cathy, FP. 307]

In Extract 8, we can identify an extended argument in terms of its components and pattern. According to Toulmin (1958), a comprehensive claim may have constituent claims in it that are considered a series of claims. This argument contains a series of claims: (1) that Malays are oppressed in Malaysia (line 1); (2) Malays accept religious oppression (line 3); (3) Islamic scholars have social power and Malays ignore this (lines 5 and 6); and (4) the situation is worsening and alarming (line 7). The arguer provides data that supports the series of claims: the Islamic education of children by their parents and the government (line 8); and that everyone is born innocent but can be brain-washed (line 9). This data to the series of claims leads to the implicit warrant (line12) that according to Simosi (2003) can be traced and completed in light of the data and backing. Thus, the implicit warrant is “a Malay child does not choose to be a Muslim by himself, rather the government and parents make the child a Muslim in the light of the definition of being Malay in the Federal Constitution of Malaysia.” The backing (line 10) strengthens the warrant by the information that Islamic laws and education are specified for Malays but other nationalities are free from religious oppression. A rebuttal is a statement that limits the generalizability of the claim or informs of exceptions to the claim, by including *other-side* information, helping the arguer maintain the impression of neutrality and avoid so-called *myside bias* (Wolfe et al., 2009). This extended argument offers more than one rebuttal; the first rebuttal (lines 11 and 12) limits the strength of the claim that Malays are religiously oppressed (line 1) by saying that Malays have no problem with being trapped by the constitutional definition of a “Malay” that conditions them to be a Muslim in Malaysia. The second rebuttal (line 14) strengthens the first rebuttal by addressing the issue of free choice. Here the arguer assumes the possibility of free choice and suggests that if Malays were left free to choose Islam without the fear of losing “Malay” status that is constitutionally linked to Malay identity, and they still chose Islam, the free-thinkers would respect that. The third rebuttal (line 15) informs of the limitations of the second rebuttal. Erduran et al. (2004) point out that an earlier presented limitation of a rebuttal strengthens the argument. In this way, the previous rebuttal serves as the claim for the current rebuttal. The second rebuttal in this argument (line 14) serves as the claim for the third rebuttal when lines 14 and 15 are read in concert. Those free thinkers who would respect Muslims (line 14) become a claim for the rebuttal ‘if they respect others freedom of choice' (line 15).

In the argument-level scheme proposed by Erduran et al. (2004), a pattern of extended argument with more than one rebuttal is a Level 5 argument. The qualitative strength of such an argument stands way above all the other levels of argumentation. The components derived and the identified pattern of argument in Extract 8 measure up to Level 5 argumentation. This indicates that the argument presented in Extract 8 is the most potent form of argument in the argument level schema.

## Conclusion

Social media provides a space to express one's view of a particular topic freely. People who feel marginalized such as former Muslims and atheists in Muslim majority countries can have the opportunity as social media affords to share and express their views. They use language strategically in their postings to construct arguments that challenge Islamic authoritative discourse and to explain their renunciation of the religion. The analysis shows that the arguments identified in the ex-Muslims' postings challenge several Islamic teachings of which the most popular topics are God as the creator; Muhammad as a prophet; Quran as a perfect book; women rights in Islam; Islam as a religion of peace and harmony; life hereafter; the call for prayer and fasting during Ramadan, and blasphemy. The arguments presented in their social media postings vary in terms of structure and strength. Using Toulmin (1958) model of argumentation to analyze the participants' postings above has illustrated the variety of argument structures employed in the postings, the main ones being: C (claim), CDW (claim, data, and warrant), and CDWB (claim, data, warrant, and backing). The least used argument structures include CDWBR (claim, data, warrant, backing, and rebuttal) and CDWBQR (claim, data, warrant, backing, qualifier, and rebuttal). The persuasiveness of the participants' argumentation was analyzed using Erduran, Simon and Osborne (2004) argument-level scheme, categorizing the arguments into five levels, ranging from Level 1 to Level 5. Most of the arguments were at Level 2 (CDW and CDWB). The next most common arguments measured were Level 1 (C), then Level 3 (CDW R [weak rebuttal] and CDWB R), Level 4 (CDWBR and CDWBQR [one strong rebuttal]), and Level 5 (CDWBQR [more than one rebuttal]). The majority of the argumentation was at the lower levels (Levels 1, 2, and 3), i.e., with weaker levels of argumentation in the participants' postings. The strongest argument levels (Levels 4 and 5) had fewer identified arguments from the research data due to the technical limitations of micro-blogging on Twitter. Extended argumentation was found only on Facebook postings as Twitter posts have a 40-word limit, possibly why fewer Level 4 and Level 5 arguments were found in the research data overall. Hence, in this study, the limitations and generalizability of results are partly due to the varied nature of social media platforms and the amount of space they offer to the users; second, the argumentation strength could be investigated in a more sophisticated way by including comments on the postings, which are not the part of data due to the demarcation of only Malaysian participants in this study. Finally, the dimension of the discursive construction of former Muslims' argumentation does not fall in the scope of this study that can provide new insights into the strength of argumentation in this study.

Though some social media data carry the risk of inauthenticity, in this study, the authenticity of data can be argued as the recruited participants do not hide their identity and are in the limelight. Furthermore, using the snowball technique further confirmed Malaysian participants were recruited. The strength of this study can also be argued in terms of the authenticity of data due to the avoidance of the participants' potential desirability of postings for this study as this study collected back-dated postings; second, the strength of this study also emerges from the importance of social media against the state censure policy as social media has no power to censure the postings that provided more expressive data as compared to offline expressions related to the religion in a Muslim majority country.

### Contribution

Social media postings by former Muslims are not merely the account of expression and entertainment; rather, the postings serve as arguments through which discourse is constructed to justify the abandoning of the Muslim faith. This specific discourse demonstrates the strengths and weaknesses of argumentation on behalf of which the justifications of renouncing the religion are offered.

## Data Availability Statement

The raw data supporting the conclusions of this article will be made available by the authors, without undue reservation.

## Ethics Statement

Ethical review and approval was not required for the study on human participants in accordance with the local legislation and institutional requirements. The patients/participants provided their written informed consent to participate in this study. Written informed consent was obtained from the individual(s) for the publication of any potentially identifiable images or data included in this article.

## Author Contributions

All authors listed have made a substantial, direct and intellectual contribution to the work, and approved it for publication.

## Funding

This work was supported by the Ministry of Higher Education Malaysia under grant FRGS/1/2018/SS103/UniSZA/02/11.

## Conflict of Interest

The authors declare that the research was conducted in the absence of any commercial or financial relationships that could be construed as a potential conflict of interest.

## Publisher's Note

All claims expressed in this article are solely those of the authors and do not necessarily represent those of their affiliated organizations, or those of the publisher, the editors and the reviewers. Any product that may be evaluated in this article, or claim that may be made by its manufacturer, is not guaranteed or endorsed by the publisher.
